# Is the fine motor–executive functions link stronger for new compared to repeated fine motor tasks?

**DOI:** 10.1371/journal.pone.0241308

**Published:** 2020-11-05

**Authors:** Michelle N. Maurer, Claudia M. Roebers

**Affiliations:** Department of Developmental Psychology, Institute of Psychology, University of Bern, Bern, Switzerland; University of Oviedo, SPAIN

## Abstract

Although the motor–executive function (EF) link is actively being investigated, there remain open questions surrounding why some studies found associations between specific motor and specific EF tasks, while others did not. Furthermore, it is also yet unknown which factors impact the magnitude of the motor–EF link. Findings from neuroimaging studies have proposed that neural activity in networks that are important for motor and cognitive tasks is especially strong when a task is new. In the present behavioral study, we systematically investigated the impact that task novelty had on the motor–EF link. In our study, n = 124 kindergarten children aged five to six administered in a within-subject design three fine motor tasks of the Movement Assessment Battery for Children-2 (Posting Coins, Threading Beads, and Drawing Trail) twice in succession (new vs. repeated), and three EF tasks (adapted versions of a Flanker, a N-back, and the Advanced Dimensional Chance Card Sort task). Results not only replicated the fine motor–EF link, but also showed a significantly stronger association between EF and the new task compared to the repeated Drawing Trail task. However, for the time-based task of Posting Coins and Threading Beads, motor–EF associations did not differ between the new task and the repeated task. Future investigations of more than two repetitions will provide further insights into the assumption that the motor–EF link is mainly driven by the EF processes triggered when a task is new, demands attention, and requires fast and flexible adaptation.

## Introduction

Research on the association between motor and cognitive functions in children has rapidly grown in the last years [1]. This interest can partly be explained by the fact that specific motor and cognitive functions, particularly executive functions (EF), play a predictive role in school readiness and later academic achievement, both concurrently and longitudinally [2, 3]. Although the motor–EF link is widely acknowledged nowadays, there are studies that have failed to find evidence for this link [4]. For instance, one review of previous investigations into the motor–cognition link clearly showed that this interrelation is not as well-established nor as robust as is sometimes assumed [5]. This conclusion is illustrated by studies that found only a weak motor–EF link, or no link at all. Moreover, when interrelations between motor and cognitive tasks were found, these associations were typically very task specific. Given the debate over the motor–EF link, it is not surprising to see that little is known about which factors impact the link. In the present contribution, we therefore aim to further understand the motor–EF link by addressing the impact that task novelty has on this link. This study thus aims to tackle the assumption that the fine motor–EF link is stronger for new fine motor tasks compared to repeated fine motor tasks. This assumption can be illustrated in the following example.

Imagine a child who is first confronted with a specific motor task to do, such as rolling a ball into a bucket located at a predefined distance from the child. The very first trials will be characterized by trying to find out how much force is needed, how the body needs to move, when to release the ball, and then how to overcome the errors made in the previous attempts. With trial and error, the child will discover a strategy to use for the task and will fine-tune movement sequences. These processes lower the cognitive effort that is needed to master the task. In other words, with increasing practice, cognitive resources are freed. Cognitive functions that are especially crucial for mastering such new and demanding situations, or when learning a new skill, are known as EF.

EF is an umbrella term involving adaptive and goal-directed cognitive processes that are typically associated with activation in the prefrontal cortex [6]. According to Miyake et al. [7], EF are considered to be a unitary construct with three partially dissociable components, namely, updating, response inhibition, and shifting. Updating refers to the ability to actively manipulate information in working memory. Inhibition is required to suppress a dominant or automatic response. Finally, shifting involves switching from one rule to another.

EF are assumed to be especially critical not only when solving new and unfamiliar cognitive tasks, but also when performing new motor tasks. This may partly explain the association between motor tasks and cognitive tasks when measuring performance on tasks in both domains. The motor learning literature suggests that cognitive demands on motor performance are especially high in early stages of motor learning [8], thus it is assumed that also EF are especially involved in new motor tasks. For instance, to perform a new and challenging motor task, EF are needed to remember task instructions, to overcome automated or prepotent responses, to control movements, and to flexibly adapt to a constantly changing environment. Among various motor skills, fine and gross motor skills [9] and visual motor integration skills [10] were found to be associated with EF. Fine motor skills are typically more closely and more consistently related to EF than gross motor skills [11]. This is why fine motor skills are the focus of the present study.

One compelling line of evidence on the association between motor skills and EF stems from neuroimaging studies [12]. Such studies consistently show that brain structures like the prefrontal cortex, cerebellum, basal ganglia, and particularly the striatum, are important for motor and cognitive tasks. These brain structures and neural networks are concomitantly activated during specific motor and specific cognitive tasks. Interestingly, activation in these brain structures is especially strong when a task is new as opposed to familiar. Cognitive-attentional resources are especially needed in early stages of motor learning [13]. During this early phase, attention is mainly directed to understanding the task and to following its rules. Strategies to solve the task are discovered and tested with trial and error, leading to initially rather slow and error prone performance in tasks [13]. With practice, performance speed and accuracy generally increase while cognitive demands decrease [14].

However, what is unknown is whether and to what extent practicing two motor trials lowers cognitive demands, as indicated by a decrease of EF involvement. One possibility to investigate this question is to compare results from new versus repeated fine motor task trials within participants and then quantify the associations to EF. This was done in the present study by administering each fine motor task twice in succession. Unlike previous studies administering dozens of trials on motor tasks which are loosely related to the everyday motor experiences of a child (such as the Serial Reaction Time Task [15]), we administered two trials on motor tasks which children actually encounter in their everyday life. Thus, children should be well able to perform the tasks as they have some experience, but the specifics of the motor execution and the task affordances are new. We assumed that when the specific fine motor tasks were new, EF should be recruited more extensively than when the tasks are repeated, as first task strategies have been discovered and sequencing of sub-responses has been achieved. Although two administrations of a task may seem to be very few, there are indications that first changes in cognitive involvement may occur within the very first trials: For instance, it is known that the cerebellum can learn from a single trial [16]. Based on sensorimotor information, the cerebellum compares the outcome expected to the actual motor outcome and adapts the motor commands for following attempts [17]. Furthermore, a study in 7-year-olds found initial indications of higher correlations between motor tasks and EF in the first task compared to the second task [18]. This finding seems to suggest that EF processes are especially recruited when a task is new. While the study of Roebers and Kauer [18] did not systematically investigate the role of that task novelty, the present study experimentally manipulated task novelty by administering each fine motor task twice in succession. By doing so, the present study aims to better understand the fine motor–EF link and the impact that task novelty has on it.

## Method

Questions of the present study were investigated by using data which is part of a bigger project on children’s performance on fine and gross motor tasks of varying difficulty (reference withheld). With the different focus of the present approach, new aspects of this rich data set are being addressed. One hundred twenty-four kindergarten children (46% boys) aged 5 to 6 years (*M* = 71 months, *SD* = 5.8, range = 60–82 months) participated in the study. Data from an additional eight children were excluded due to their absence at one of the testing days (n = 4) or reported motor disabilities (n = 4). Eighty-one percent of the children were native speakers of the country’s language, and 19% had another first language. All children were sufficiently fluent in the country’s language to follow the task instructions. Prior to participation, written informed consent was obtained from the parents and verbal assent from the children. This study was conducted in line with the established ethical principles of the APA, conforming to the declaration of Helsinki, and was approved by the Ethics Committee of the Faculty of Human Sciences of the University of Bern, Switzerland (Approval No. 2013-12-733209).

### Procedure

Children were tested individually at their kindergarten school. By using a within-subject design, children were randomly assigned to four different counterbalanced task orders. Each of the three fine motor tasks was administered twice successively, with a short break of 20 s in between the two task administrations. During initial task execution, motor tasks were assumed to be new. During the second task execution, children had already experimented and familiarized with the task.

### Measures

The children did tasks to measure their fine motor skills and their EF. Fine motor skills were assessed with the Manual Dexterity subscale of the Movement Assessment Battery for Children-2 (M-ABC-2) [19]. There were three tasks. First, in the Posting Coins task, children were asked to insert 12 coins with their dominant hand, one after the other, as fast as possible, into the slot in a box. The time it took to complete the task was recorded. Right after the task was finished, the coins were repositioned again and the task was repeated. Second, in the Threading Beads task, children picked up cube beads one at a time, and threaded the beads on a lace as quickly as possible. The time it took to thread 12 beads on the lace was measured. Right after completing the task, the beads were re-positioned and the same task was repeated. Third, the Drawing Trail task required children to draw (with their dominant hand) a single, continuous line between two curvy lines, ideally without touching or crossing the boundaries. The number of times their line touched or crossed the boundaries was defined as an error, and served as a dependent measure. Again, after a short break, the task was repeated.

EF were measured in the children with three frequently used tasks, each emphasizing mainly one of the EF components: inhibition, updating, and shifting. For inhibition, in a child adapted computerized Flanker task [18, 20], five fish were presented on a screen. Children were instructed to respond as quickly and as accurately as possible to the orientation of the central fish by pressing either the right or the left response button in front of them, depending on the orientation of the central fish. In the congruent trials, the central and the flanking fish were facing the same direction, but in the incongruent trials, the central fish was facing the opposite direction. The task consisted of a congruent block followed by a mixed block containing twenty congruent and twenty incongruent trials in randomized order. We recorded the mean reaction times of the correctly solved incongruent trials and the mean accuracy of the incongruent trials.

For updating, an adapted computerized N-back task [21, 22] was used to assess the updating of working memory representations. Children were confronted with a sequence of familiar animals appearing on the screen. Each animal was presented for 1900 ms with an inter-stimuli interval of 100 ms. The length of the animal sequence varied randomly between four and seven animals. Children were asked to remember the last two animals, and to name them in the order they were presented in, as soon as a question mark appeared on the screen. After the practice block, children completed a test block of ten sequences. One point for each correctly remembered animal and one additional point for the correct order could be achieved on each trial (30 points in total). The accuracy score (percentage of points) was used as a dependent variable.

For shifting, in the Advanced Dimensional Change Card Sort (ADCCS) task [23], the experimenter presented three boxes to the children. One card was affixed to each box, depicting either a blue square, a yellow circle, or a red triangle. Children were presented cards with different shapes (square, circle, and triangle) of different colours (blue, yellow, and red). First, children were asked to sort cards by colour by putting them into the blue, yellow, or red boxes. Second, they were asked to sort cards by shape by putting them into the corresponding boxes (with the square, circle, or triangle cards on them). In the critical shifting block, children were instructed to sort twenty cards according to shape if there was a star on the card (20% of the cards), but to sort according to color if there was no star on the card (80% of the cards). The time in seconds it took to complete the task as well as the accuracy scores (percentage of correctly sorted cards) were measured.

## Statistical analyses

We analysed data using IBM SPSS Statistics 25. All dependent measures were z-standardized and inverted if necessary, so that uniformly higher values indicate superior performance. We defined scores exceeding the inter-individual mean by three standard deviations (*SD*) as outliers, and we replaced them with the value of the third *SD* (applied to 0.7% of all data points). To test the fine motor–EF link, bivariate correlations were tested. Differences between two dependent correlations were tested with a Fisher Z-test [24]. Paired-samples t-tests were used to test differences between the new and repeated fine motor tasks. The level of significance was set to *p* < .05.

## Results

### Fine motor tasks

Descriptive statistics for the new and repeated fine motor tasks are presented in Table 1. We estimated test-retest reliability coefficients between performance on the new and repeated tasks by using the Spearman-brown formula [25], which revealed that the reliability was acceptable to good. Paired-samples t-tests revealed on average that performance in the new and repeated Posting Coins tasks and Drawing Trail tasks did not differ (Posting Coins t(123) = 1.38, *p* = .17; Drawing Trail t(123) = -1.09, *p* = .28). In contrast, children performed the Threading Beads task faster the second time than the first, t(123) = 3.81, *p* < .01, *d* = .34.

**Table 1:** Mean (M), standard deviation (SD), and range for the new and repeated fine motor tasks (n = 124).

	New		Repeated		Retest-Reliability
	M (SD)	Range	M (SD)	Range	
Posting Coins (seconds)	20.42 (3.42)	14–33	20.04 (3.43)	13–33	.75
Drawing Trail (errors)	5.40 (3.15)	0–13	5.69 (3.15)	0–16	.71
Threading Beads (seconds)	45.04 (11.70)	26–87	42.40 (10.45)	24–74	.86

Spearman Brown formula used to estimate retest-reliability.

### EF

Descriptive statistics for the EF tasks are presented in Table 2. The skewness and kurtosis coefficients for the different EF variables were acceptable. Bivariate correlations were negative between performance in the accuracy-based and the time-based measures of the EF tasks (r = -.29, *p* < .01, for the Flanker task, r = -.40, *p* < .01, for the ADCCS task). This result indicated that children who responded faster were generally responding also more accurately. In other words, and as was expected in this age range of children (5–6 years), there was no speed–accuracy trade-off.

**Table 2:** Mean (M), standard deviation (SD) and range of the different EF measures (n = 124).

	M	SD	Range	Skewness	Kurtosis
Flanker (milliseconds)	1570.67	519.70	687–3067	.84	.40
Flanker (accuracy)	86.09	18.77	15–100	-1.55	1.63
ADCCS (seconds)	62.44	18.22	31–129	.99	1.57
ADCCS (accuracy)	94.21	7.24	70–100	-1.46	1.48
Animal updating (accuracy)	75.81	16.77	23–100	-.46	-.32

In the Flanker and ADCCS tasks, children were instructed to perform as quickly and as accurately as possible. Therefore, standardized scores were calculated by combining (i.e., adding) the accuracy and time-based measures. Together with the z-standardized accuracy score of the updating task, a composite EF score was built and used as a dependent measure of EF for further analyses. There were two reasons for having a combined EF measure. First, we intended to map individual differences in the different facets of EF broadly. Second, EF in young children tend to be rather undifferentiated [26], and independent contributions of the single EF components are not yet expected [27].

### The fine motor–EF link

As a first step, we tested whether our data revealed an association between the fine motor tasks and the EF tasks we administered. This association has been documented in some specific studies using specific tasks. In our study, the association between the fine motor tasks and EF tasks was first tested on a general level. Specifically, we combined results for fine motor performance across both the new and repeated tasks, and also across all three fine motor tasks. Our results showed that overall fine motor performance correlated with EF to r = .42, *p* < .001, which indicates a medium effect [28]. This finding indicates that children who performed well on the fine motor tasks generally also tended to perform well on the EF tasks, and vice versa.

### The impact of task novelty on the fine motor–EF link

As a next step, the research question was addressed to see whether the fine motor–EF link differs in magnitude for the new fine motor tasks compared to repeated fine motor tasks. The new fine motor tasks combined correlated with EF to r = .46, *p* < .001, and the repeated fine motor tasks correlated with EF to r = .38, *p* < .001. Despite the descriptively higher associations for the new compared to the repeated fine motor tasks, the correlation coefficients did not differ significantly from each other, *z* = 1.30, *p* = .19.

In order to have a more fine-grained picture of the impact that task novelty had on the fine motor–EF link, correlations for the new tasks and repeated tasks were tested for each fine motor task separately. Correlation coefficients are depicted in Table 3. Results revealed that for the Posting Coins task and the Threading Beads task, the correlations with EF did not differ significantly for the new and repeated tasks in both cases (*z* = -0.41, *p* = .69, for Posting Coins; *z* = 0.84, *p* = .40, for Threading Beads). However, the correlation coefficient between the new Drawing Trail task and EF (r = .41) was significantly stronger than the correlation coefficient between the repeated Drawing Trail task and EF (r = .25), *z* = 2.01, *p* = .04.

**Table 3:** Pearson correlations between EF and each new and repeated fine motor task.

Measures	Posting Coins		Drawing Trail		Threading Beads	
	New	Repeated	New	Repeated	New	Repeated
**EF**	.29**	.32**	.41**	.25*	.32**	.27*

*p < .05

**p < .01 (2-tailed).

## Discussion

The present study was designed to experimentally address the impact of task novelty on the association between fine motor skills and EF in 5- to 6-year-old kindergarten children. Overall, our results revealed significant interrelations between performance on fine motor and EF tasks. These results are in line with those from previous studies [9, 11, 29]. In order to explore the impact that task novelty had on the motor–EF link, each fine motor task was administered twice in relatively rapid succession. While in the first round, tasks and their affordances were assumed to be new, in the second round, children had already gained familiarity with the first tasks.

Overall, our results showed that the correlation between EF and fine motor skills was not much different in the new fine motor tasks (r = .46) compared to the correlation between EF and the repeated fine motor tasks (r = .38). While individual differences in new fine motor tasks and EF shared about 21% of their variances, the shared variances of individual differences were 14% for the repeated fine motor tasks and EF, which is slightly less. Although each fine motor task was only performed twice, descriptively lower correlations were found for two out of three repeated tasks. These lower correlations might indicate the beginning of a decline of EF involvement with continuing motor practice.

As for specific fine motor tasks, a significantly higher correlation was found between fine motor skills and EF in the new Drawing Trail task compared to the repeated task, even though children did not perform significantly better in the repeated Drawing Trail task. As assumed, initial performance on the Drawing Trail task demanded extensive proprioceptive and visual-motor coordination, as well as planning and sequencing processes. These processes may have involved how to hold the pencil, how to adjust pen pressure, and how to control finger and hand movements in order to navigate the line between the given boundaries. In addition, drawing speed needed to be adjusted constantly to optimally achieve the task’s goal. It is reasonable to assume that these processes may have contributed to a decline of EF engagement during the repeated Drawing Trail task. This is because performance became more fluent and less dependent on cognitive resources in the repeated task compared to the new task.

Our findings are in line with models of motor learning, which suggest that with continuing motor practice, cognitive demands decrease [8, 30]. According to these models, the most rapid changes of motor performance and cognitive involvement happen at the very beginning of the learning process. Although children did not improve performance in every fine motor task, the results suggest that only after two trials in our Drawing Trail task, an initial decline of EF involvement can be seen. Likewise, findings of neuroimaging studies repeatedly showed that neural activation decreases with practice in networks that are crucial for motor and cognitive functions [31].

Furthermore, this fast decline in EF involvement can partly be explained by the cerebellum’s role in processing sensorimotor feedback in fast error-based learning [32]. It can be assumed that for the Posting Coins task and for the Threading Beads task, which both are speed-based tasks, the sensorimotor feedback provided to the cerebellum was subtler compared to that in the accuracy-based Drawing Trail task. Consequently, error correction and adaptations of the internal models of the cerebellum [16] probably require more extensive practice until EF involvement really starts to decrease. This interpretation could partly explain why fine motor skills in the new Posting Coins task and the new Threading Beads task were not associated more strongly with EF than in the repeated tasks. However, further research addressing EF involvement across more than two repeated trials is needed to better understand children’s cognitive involvement during specific motor tasks. In addition, further task characteristics as well as individual factors that may impact the motor–EF link need to be addressed in future research.

In sum, the present study provides first hints that even after only two trials, the magnitude of the motor–EF link may decrease. Results of the Drawing Trail task indicate that EF might be especially involved when the task is new compared to repeated. With increasing task familiarity and practice, children are likely already familiar with the specific task affordances, have discovered strategies and the sequencing of sub-responses has been achieved. This may lead to a decrease in cognitive involvement with continuing motor practice, even though motor performance did not improve, which was the case for the repeated Drawing Trail task. In addition, our findings point to the importance of further investigating the impact that task novelty has on the motor–EF link when studying more than two trials. Such research can help us better understand the inconsistent findings reported in the literature.

## Supporting information

S1 Dataset Dataset underlying the findings of this study. (XLSX)
